# Two new species and a new species record of *Aglaia* (Meliaceae) from Indonesia

**DOI:** 10.3897/phytokeys.155.53833

**Published:** 2020-08-07

**Authors:** Caroline M. Pannell, Jan Schnitzler, Alexandra N. Muellner-Riehl

**Affiliations:** 1 University of Oxford, Department of Plant Sciences and Daubeny Herbarium (FHO), South Parks Road, Oxford OX1 3RB, United Kingdom Leipzig University Leipzig Germany; 2 Royal Botanic Gardens, Kew (K), Richmond, Surrey TW9 3AE, United Kingdom University of Oxford Oxford United Kingdom; 3 Queen’s University Belfast, Marine Laboratory, 12–13 The Strand, Portaferry, County Down, BT22 1PF, United Kingdom Royal Botanic Gardens Kew United Kingdom; 4 Leipzig University, Institute of Biology, Department of Molecular Evolution and Plant Systematics & Herbarium (LZ), Johannisallee 21–23, D-04103 Leipzig, Germany Queen’s University Belfast United Kingdom; 5 German Centre for Integrative Biodiversity Research (iDiv) Halle-Jena-Leipzig, D-04103 Leipzig, Germany German Centre for Integrative Biodiversity Research Leipzig Germany

**Keywords:** *
Aglaia
*, conservation status, Indonesia, Meliaceae, phylogeny, taxonomy

## Abstract

Two new species of *Aglaia* from Indonesia are described, *Aglaia monocaula* restricted to West Papua, and *Aglaia nyaruensis* occurring on Borneo (Kalimantan, Brunei, Sabah and Sarawak). A phylogenetic analysis using nuclear ITS and ETS, and plastid *rps15-ycf1* sequence data indicates that the two new species of *Aglaia* are also genetically distinct. *Aglaia monocaula* belongs to sectionAmoora, while *A. nyaruensis* is included in section Aglaia. A dichotomous key, drawings and three-locus DNA barcodes are provided as aids for the identification of the two new species of *Aglaia*. In addition, the geographic range of *Aglaia mackiana* (section Amoora) is expanded from a single previously known site in Papua New Guinea to West Papua, Indonesia.

## Introduction

The classification of the family Meliaceae continues to be refined (Muellner et al. 2003, 2005, 2009a, b, Muellner and Mabberley 2008, Pennington and Muellner 2010, Köcke et al. 2015, Clarkson et al. 2016, Gama et al. 2020) and new taxa are still being discovered and described (e.g., Pannell 2004, 2019). *Aglaia* Lour. is the largest genus of the family, and, with at least 120 arborescent species, presents more taxonomic problems in species delimitation than any other genus of the family (Pannell 1992, 1995, 1998a, b, 2004, Muellner et al. 2005, 2008a). *Aglaia* forms an important component of the moist tropical forest in the Indomalesian region. The distribution range comprises the tropics of southeastern Asia from Sri Lanka and India to Australia (Queensland, Northern Territory, and Western Australia) and eastwards to the island of Samoa in Polynesia and north to the Mariana (Saipan, Roti, and Guam) and Caroline Islands (Palau and Ponape) in Micronesia (Pannell 1992). A monograph of the entire genus throughout its range has been published (Pannell 1992), of the Malesian species, including New Guinea, in Flora Malesiana (Pannell 1995), and of the Bornean species in the Tree Flora of Sabah and Sarawak (Pannell 2007).

From the 1990s, the genus received increasing scientific focus due to its bioactivity potential (Muellner et al. 2005, and references therein), including inhibiting activity against Ebola-, corona-, Zika-, Chikungunya- and hepatitis E-viruses (Müller et al. 2018, 2020). A recent research call in the field of “Biodiversity and Health”, funded by the German Federal Ministry of Education and Research, has led to a surge of interest in the taxonomic investigation of plant groups of potential interest for the future development of new antiinfective compounds. A taxonomic survey of *Aglaia* in Indonesia in the course of this research program has led to the discovery of two new species. We here describe these two new species from Indonesia. In addition, we report a new record of *Aglaia* from Indonesia, previously known only from Papua New Guinea.

## Materials and methods

### Morphology

The two new species are described based on field observations and examination of herbarium specimens at BO, FHO, K, L, and A, using morphological characters that distinguish them from all other species in the genus *Aglaia*. Descriptions were written from herbarium specimens. Measurements were made with a tape-measure and calipers. The structure of the indumentum and its distribution was observed and described under a dissecting microscope at magnifications of more than 20×. Flowers were rehydrated by boiling in tap water. They were placed on a glass slide covered with 1 mm graph paper for scale and dissected, measured and described under a dissecting microscope. Additional information on locality, habitat, ecology, plant form, bark and wood characters and fruits was collected in the field and taken from herbarium labels. Conservation threat assessment followed IUCN Categories and Criteria (IUCN 2012).

### DNA extraction, amplification, and sequencing

Total genomic DNA was extracted for representative samples of each species of *Aglaia* described herein (Table 1) using a Macherey-Nagel NucleoSpin Plant II kit. The protocol was modified by adding 40 ul β-mercaptoethanol and 2% polyvinylpyrrolidone (PVP). ITS was amplified either as a whole using the primer combination 17SE_m/26SE_m (Grudinski et al. 2014) or, if this failed, adding two internal primers (F1_ITS/R1_ITS, Muellner et al. 2005) to amplify the first and second part of the ITS region separately. ETS was amplified using the primers 18S_ETS (Baldwin and Markos 1998) and a newly designed primer, 18S_MEL [5’-GTG TGA GTG ATT GGA T-3’; this study]. The plastid region *rps15-ycf1* was amplified using the primer pair rps15-IGSR/ycf1-IGSR (Prince 2015).

For all amplifications, we used the Phusion High-Fidelity DNA Polymerase (New England BioLabs, Ipswich, MA, United States) according to the manufacturer’s protocol. Annealing temperature for ITS (whole region or in two parts) and ETS was 51.5 °C, and for *rps15-ycf1* 51 °C. PCR products were cleaned using the NucleoSpin Extract II Kit (Macherey-Nagel, Düren, Germany). Sequencing reactions and analyses were run by LGC Genomics (Berlin, Germany).

All sequences were assembled and edited using Geneious (v7.06, Kearse et al. 2012). Consensus sequences were aligned using MUSCLE (v.3.8.31 Edgar 2004) as implemented in Geneious, and all alignments were thoroughly checked and further refined manually. For ITS, sequences were explored for the presence of several structural motifs, allowing for the detection of pseudogenes: the conserved angiosperm motif GGCRY–(4 to 7n)–GYGYCAAGGAA (Liu and Schardl 1994); the conserved (C1–C6) and variable (V1–V6) domains determined for plant ITS2 sequences (Hershkovitz and Zimmer 1996); and the conserved angiosperm motif 5´-GAATTGCAGAATCC-3´ within the 5.8S rRNA gene (Jobes and Thien 1997). Secondary structure predictions were confirmed by hemi-compensatory base changes and full compensatory base changes that preserved the predicted folding pattern. Sequences for the new species were deposited in GenBank (http://www.ncbi.nlm.nih.gov/; Table 1). Voucher information, geographic origin, and GenBank accession numbers for all samples included in this study are provided in Suppl. material 1: Table S1.

### Phylogenetic analyses

Newly generated sequences of ITS were combined with the data from Muellner et al. (2008b) and an improved and reduced version of the data matrix used in Grudinski et al. (2014), which included representatives of all sections of *Aglaia* and outgroups. The best-fit model of nucleotide substitution, as determined using the Akaike information criterion (AIC) in jModelTest 2.1.10 (Darriba et al. 2012), was GTR+G for ITS and *rps15-ycf1*, and GTR+G+I for ETS. Phylogenetic analyses for each individual marker and combined datasets (both nuclear markers, all markers) were performed using MrBayes v3.2.7 (Huelsenbeck and Ronquist 2001, Ronquist and Huelsenbeck 2003) with four runs (six Markov chains each) for 20–25 million generations (depending on the marker), sampling every 10,000 steps. In the combined analyses, datasets were partitioned according to the genetic markers with model parameters being unlinked across the partitions. Efficient chain mixing and convergence of the runs to the same posterior distribution, as well as the adequacy of sampling (using the Effective Sample Size [ESS] diagnostic) were evaluated by examining the log files in Tracer v1.7 (Rambaut et al. 2018). For each analysis, a majority-rule consensus tree was constructed after excluding the first 20% of samples as burn-in.

## Results and discussion

### Taxonomy

**Section Amoora**

#### 1. Aglaia monocaula

Taxon classificationPlantaeSapindalesMeliaceae

Pannell sp. nov.

382A9D7B-DFDD-5EE6-B523-8D068DC4F31D

urn:lsid:ipni.org:names:77210863-1

Fig. 1

##### Diagnosis.

***Aglaia monocaula*** resembles *Aglaia flavida*, from which it differs through being a smaller, unbranched tree with reticulation subprominent and no indumentum on the lamina of the lower surface of the leaflets. It is unique in the genus in having a dark blackish-brown, slightly swollen, region at the base of the petiolules.

**Figure 1.**
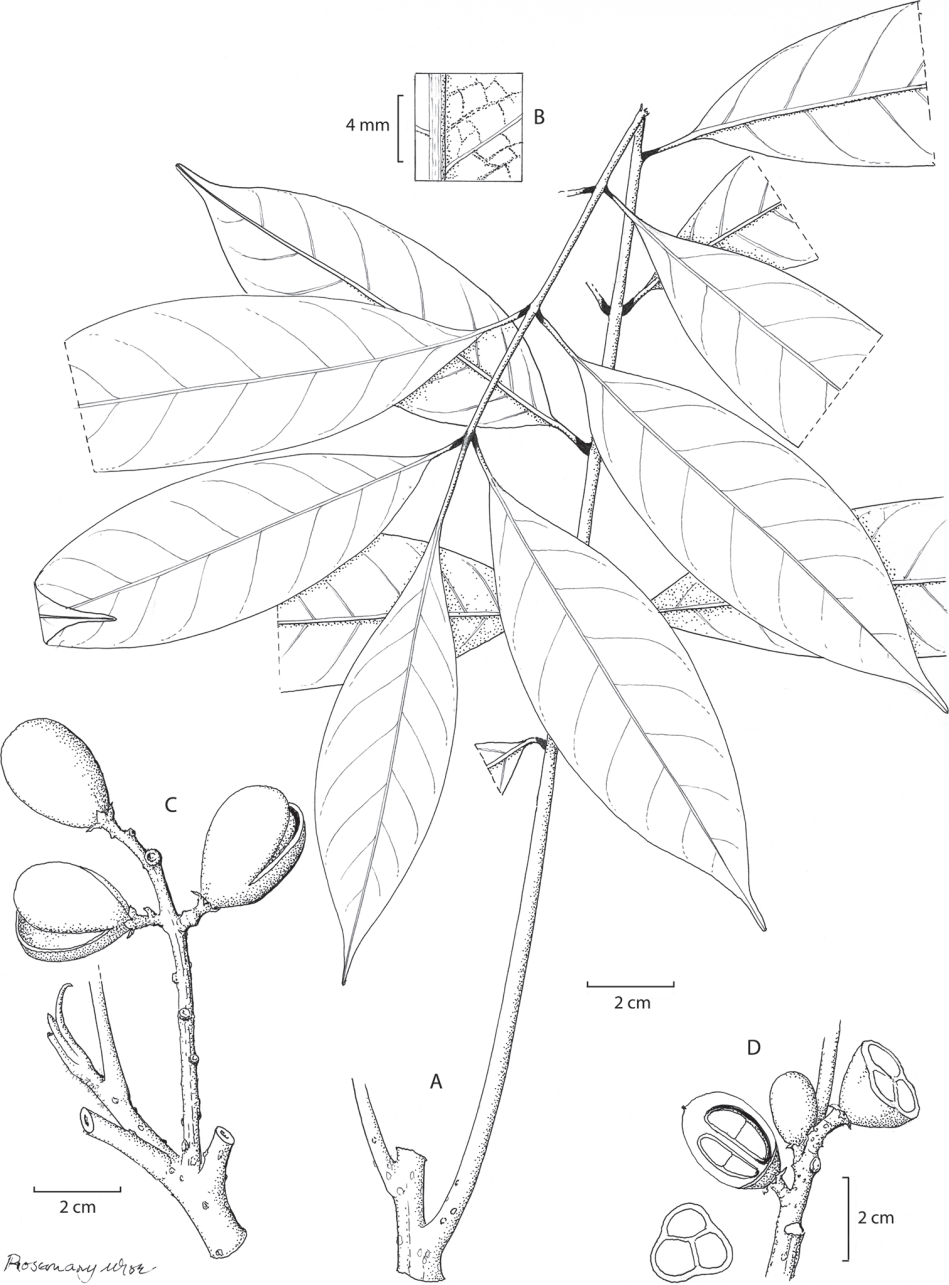
*Aglaia monocaula* Pannell **A** habit **B** detail of lower surface of the leaflet **C** apical shoot subtending infructescence in a leaf axil **D** part of the infructescence with fruits cut transversely to show the three seeds and longitudinally to show the junction between the two peltate cotyledons typical of the genus *Aglaia* (Drawn by Rosemary Wise, edited by Alexandra Muellner-Riehl).

##### Holotype.

Indonesia. **West Papua**: Kecamatan Ayfat, neighborhood of Ayawasi, fr. 12 Feb. 1995, *K. Yumte* 126 (L)

Tree, 3–10 m high, *unbranched*, with a terminal tuft of spirally inserted leaves; bole 4 cm in diameter; latex white. Twigs greyish-brown with large orange-brown pustules, densely covered with orange-brown and dark brown compact stellate hairs at the apex, glabrescent on older wood.

Leaves 47–70 cm long, 28–32 cm wide; petiole 11–30 cm long; the petiole, rachis and petiolules with few to numerous hairs like those on the twigs, glabrescent. Leaflets 15, the laterals opposite or subopposite, coriaceous, lamina 7–16 cm long 2–5.5 cm wide, elliptical, slightly up-curved at the margins, cuneate at the slightly asymmetrical base, tapering to an acuminate apex, the acumen obtuse and 10–12 mm long; lateral veins 5–14, ascending and curved upwards near the margin, not anastomosing, lateral veins and reticulation subprominent; midrib prominent below with sparse stellate scales, absent from lower leaflet surface, upper and lower leaflet surfaces minutely rugulose; petiolules 10–15 mm on lateral leaflets, slender, 20–40 mm long on terminal leaflet, all with a dark blackish-brown, slightly swollen, region at the base of the petiolules.

Inflorescences not seen.

Infructescence 11 cm long, 7 cm wide; peduncle 6 cm long, the peduncle and fruit stalks with few to numerous hairs like those on the twigs, glabrescent. Fruits 2.8 cm long, 1.8 cm wide, ovoid, pericarp bright scarlet or pinkish-red, inner pericarp white, dehiscent with three locules each containing 1 seed; seed white where attached to the central axis of the fruit by a large hilum, aril orange.

##### Distribution.

Known only from the area around Ayawasi village in West Papua.

##### Ecology.

Primary open forest on limestone ridge to 600 m, with an abundant growth of moss. Fruits eaten by kuskus.

##### Use.

Wood used for house beams.

##### Vernacular.

sapa sai (K.Yumte)

##### Etymology.

The specific epithet of *Aglaia monocaula* refers to the unbranched habit of this small tree.

##### Conservation.

This species is known from only two fruiting specimens collected near Ayawasi village and is therefore assessed to be Data Deficient (provisional). Further collecting and monitoring is necessary to allow more conclusive estimations about the rareness and vulnerability of the species. However, the collections seen were made 24 and 25 years ago, so the likelihood of obtaining further material from this species is not great.

##### Additional specimen.

Indonesia. **West Papua**: top ridge of limestone hills south of Ayawasi village, fr., 1 May 1996, *Polak* 1221 (FHO)

##### Notes.

This new species is represented by two fruiting specimens of monocaul trees that have leaves with a long petiolule on the terminal leaflet.

#### 

**Section Aglaia**

##### 2. Aglaia nyaruensis

Taxon classificationPlantaeSapindalesMeliaceae

Pannell sp. nov.

F4D12F78-50B1-5932-A173-3EF82A828A62

urn:lsid:ipni.org:names:77210864-1

Fig. 2

###### Diagnosis.

*Aglaia nyaruensis* resembles *A. foveolata*, from which it differs in its smooth leaflet lower surface, with the lateral veins and reticulation not prominent. These characters, combined with numerous pits on the leaflet upper and lower surfaces, make this species unique in the genus.

**Figure 2.**
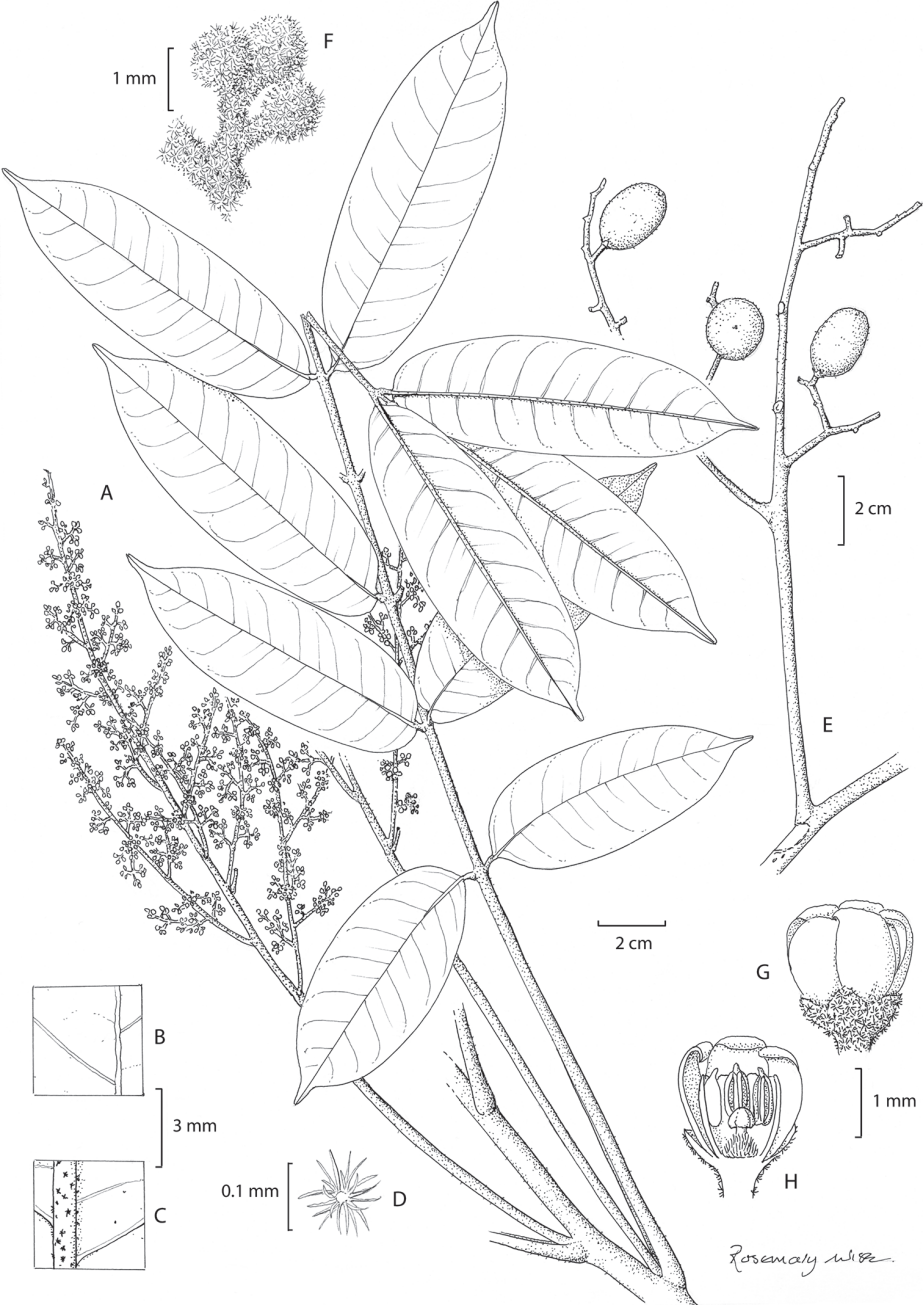
*Aglaia nyaruensis* Pannell **A** habit with male inflorescence **B** detail of upper leaflet surface **C** detail of lower leaflet showing distribution of indumentum **D** stellate hair **E** immature infructescence **F** male flower buds, densely covered with stellate hairs **G** male flower **H** half male flower (Drawn by Rosemary Wise, edited by Alexandra Muellner-Riehl).

###### Holotype.

Indonesia. **Kalimantan**: Central, Nyaru Menteng Arboretum, off km 28 road to Sampit, alt. 50m, fl., 28 Jan 1995, *K. Sidyasa* with *Ambriansyah*, *Arifin & Priyono* 1422 (holotype BO; isotypes K, L).

Tree, 10–22 m high, bole 8 m, diameter 20 cm, outer bark smooth, greyish or greyish-brown, shallowly fissured and lenticellate, inner bark pink, brownish-green or brown and fibrous, sapwood pale yellow, heartwood white; latex white or absent. Young twigs densely covered with reddish-brown stellate hairs and scales.

Leaves 25–31(–60) cm long, 14–21(–34) cm wide; petiole 7–9 cm long, the petiole, rhachis and petiolules densely covered with reddish-brown stellate hairs. Leaflets 11–15, the laterals subopposite, lamina 5.5–11.0 cm long 2.5–4.0 cm wide, narrowly oblong or elliptical, pale brownish-green when dry, rounded to cordate at the asymmetrical base, acuminate at apex with the acute acumen narrow and to 15 mm long; lateral veins 10–15, ascending and curved upwards near the margin, anastomosing some distance from the margin and with further reticulation between this and the margin of the leaflet, with shorter lateral veins in between. Midrib prominent below, lateral veins and reticulation not raised and barely visible in dried leaflets, the midrib on the upper surface of leaflet with numerous pale brown stellate hairs and scales, the midrib on the lower surface densely covered with reddish brown stellate hairs and scales, numerous on the lower leaflet surface when young, glabrescent, becoming sparse on the mature lamina near the midrib and absent from the rest of both surfaces of the lamina, with numerous pits on both surfaces; petiolules to 3 mm long on lateral leaflets to 10 mm long on terminal leaflet.

Male inflorescence 20 cm long, 10 cm wide; peduncle 8–9 cm long, the peduncle, rachis and first branches densely covered with reddish-brown stellate hairs and scales; higher orders of inflorescence branches with numerous reddish-brown stellate hairs and scales. Male flower 2 mm long, 2 mm wide; pedicel 1 mm long, the calyx and pedicel densely covered with reddish-brown stellate hairs and scales; calyx cup-shaped, deeply divided into five rounded lobes, petals 5, 1.75 mm long, 1 mm wide, yellow, obovate; staminal tube 1.5 mm long, 1.5 mm wide, obovoid with a wide mouth 1.5 mm across, anthers 6, 0.75 mm long, 0.25 mm wide, inserted half way down the tube inside and protruding through the aperture; ovary 0.5 mm long, 0.5 mm wide, ovoid, densely covered with brown stellate hairs and scales on the outside, with two locules each containing one vestigial ovule. Female flowers not seen.

Infructescence 24 cm long, 26 cm wide, peduncle 8 cm; peduncle rachis and branches densely covered with reddish-brown stellate hairs and scales. Young fruits 2 cm long, 1.5 cm wide, ellipsoid, reddish-orange, densely covered with reddish-brown stellate hairs and scales.

###### Distribution.

One record each from Kalimantan, Brunei, Sabah and Sarawak.

###### Ecology.

Peat swamp forest, swampy forest on white sand, on ultrabasic soil or on yellow-brown sandy soil over Tertiary clays, with deep litter and abundant humus and living roots. Altitude to 400 m.

###### Vernacular name.

Jalongan sasak (Bejang b. Sitam).

###### Etymology.

The specific epithet of *Aglaia nyaruensis* refers to the type locality, Nyaru Menteng in Kalimantan.

###### Conservation (provisional).

This species is known from one locality each in Kalimantan, Brunei, Sabah and Sarawak and is therefore considered to be Vulnerable.

###### Additional specimens.

Malaysia. **Sarawak**: Sibu, Haman Forest Reserve, c. 3 m alt, fr 18 June 1958, *Bejang b. Sitam* 9169 (K); **Sabah**: Sandakan, Bt Tawai Forest Reserve, 400 m alt., young flowers 26 June 1996, *S. Diwol & L. Madani* SAN 135187 (K). Brunei, **Belait**: Sungai Liang, Andalau Forest Reserve Compartment 5, 4°38'41"N, 114°30'20"E, 30 m alt., sterile, 8 March 2004, *A.N. Muellner*, *C.M. Pannell*, *G. Challen*, *Jangurun*, *Muhd Yussof*, *Ibrahim* ANM2039 (K).

#### 

**New record for West Papua, Indonesia**

##### Aglaia mackiana

Taxon classificationPlantaeSapindalesMeliaceae

Pannell, Kew Bull. 52(3): 715. 1997.

8DAD29C7-DA01-5095-9C49-B54B5546D8DC

Fig. 3

###### Remark.

Previously known only from the type locality in Papua New Guinea, this tall tree species in sectionAmoora, has the largest fruits recorded for the genus *Aglaia*. Collections from West Papua are of immature fruits and flower buds.

**Figure 3.**
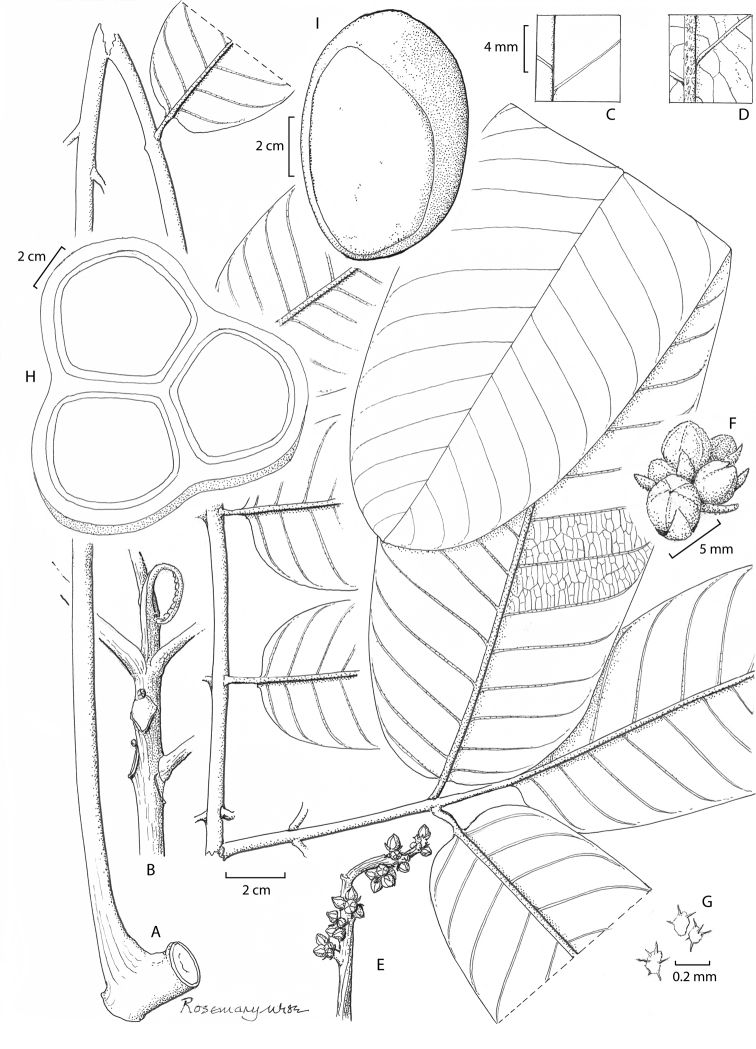
*Aglaia mackiana***A** leaf with attachment to twig **B** apex of shoot **C** detail of upper leaflet surface **D** detail of lower leaflet surface **E** immature inflorescence **F** flower buds **G** peltate scales **H** transverse section of immature fruit with three seeds **I** seed, with large hilum and intact aril (Drawn by Rosemary Wise, edited by Alexandra Muellner-Riehl).

###### Distribution.

Indonesia, two records from West Papua. In Papua New Guinea, known only from the type locality in Chimbu Province.

###### Ecology.

Primary lowland forest on the coastal plain and to 450 m altitude; canopy 25–45 m high; associate species include *Celtis*, *Sterculia*, *Pometia*, *Ficus*, *Oncospermum*, and sundry Rubiaceae. Canopy tree to 45 m tall, branching above; bole c. 1 m diameter, buttressed below; bark tan, smooth, somewhat round flaky; fruits 12–16 cm diameter, light brown, lactiferous, 3-lobed. In Papua New Guinea, the fruit either dehisces on the tree and the seeds fall to the ground or the whole fruit falls from the tree and dehisces on impact with the ground. The seeds are swallowed whole by the Dwarf Cassowary and defaecated at up to 1000 m from the parent trees (Mack 1995a, b; Pannell 1997). A fruit bat (probably *Dobsonia moluccensis*) carries seeds shorter distances, reportedly less than 100 m, away from the parent tree. Germination is semi-hypogeal, within a few days of deposition of seeds; the two large cotyledons persist at ground level for up to two years after germination.

###### Vernacular.

‘sapa peka’ (Wanda Ave 4394)

###### Etymology.

Named after Andrew Mack, who discovered this species in the course of his field work on the Dwarf Cassowary.

###### Conservation.

This species is known from only three localities, two in Papua and one in Papua New Guinea. It is therefore assessed to be Data Deficient (provisional). Further collecting and monitoring is necessary to allow more conclusive estimations about the rareness and vulnerability of the species. However, the collections seen were made 24, 25, 27, and 28 years ago, so the likelihood of obtaining further material from this species is not great.

###### Additional specimens.

Indonesia. **West Papua**: surroundings of Ayawasi, 1°09'S, 132°12'E, c. 450m, fruit, 30 April 1996, *Wanda Ave* 4394 (L); Sarmi, coastal plain, 1–3 km N of Sewan on the Waske River. 2°4'S, 138°46'E, 10–20 m, fr., 3 June 1993 *McDonald and Ismail* 3786 (BO, L, K). Papua New Guinea. **Chimbu Province**: Crater Mountain Biological Research Station, 145043–45'S, 6'05–58'E, leaves only, 1992, Mack 699 (FHO! holotype); same locality, ?1995, fruit only, Ross Sinclair RS 105 (FHO!); same locality, seeds only, 18 Aug. 1995, Mack s.n. (FHO!); same locality, fallen male inflorescences only, no date, Mack 297 (A):

### New species inserted into a truncated version of the existing key to the species of *Aglaia* in Malesia

To accommodate the three species the following couplets (in bold, labelled (i) and (ii), can be inserted into the existing key to Malesian *Aglaia* by Pannell (1995, pp. 201–212)]

#### 

**Table:** 

1a	Leaf always a single blade	**2**
1b	Leaves trifoliolate or imparipinnate	**7**
7a (1)	Leaflets with few or no hairs or scales on the lower surface, the reticulation continuous and subprominent on one or both surfaces	**8**
7b	Leaflets with at least some scales or hairs on the lower surface, although these may be few and difficult to see, reticulation not continuous and subprominent on either surface, or if subprominent, then with indumentum on lower surface of leaflet	**10**
8a (7)	Leaflets with reticulation subprominent on the lower surface	**8a (i) & (ii)**
8a(i)	Tree branched, leaflets 2–7, fruits indehiscent	**66. *A. cumingiana***
8a(ii)	Tree unbranched, leaflets 15, fruits dehiscent	***A. monocaula***
8b	Leaflets with reticulation subprominent on both surfaces	**9**
10a (7)	Leaflets linear-lanceolate or narrowly elliptical, most being at least 5 times longer than wide	**11**
10b	Leaflets ovate, elliptical, oblong, obovate, lanceolate or oblanceolate, most less than 5 times longer than wide	**14**
14a (10)	Indumentum dense, of white or pale brown hairs or scales which totally conceal the lower surface of leaflet	**15**
14b	Indumentum reddish-brown or, if pale, not totally concealing the lower surface of leaflet	**20**
20a (14)	Lower surface of leaflet so densely covered with reddish-brown or orange brown hairs or scales, that the surface is not or barely visible between them	**21**
20b	Hairs or scales absent from the lower surface or, when present, the lower surface of leaflet readily visible between them	**24**
24a (20)	Indumentum of peltate scales, sometimes with stellate scales interspersed	**25**
24b	Indumentum of stellate hairs or scales; peltate scales absent	**69**
25a (24)	Indumentum of peltate scales only	**26**
25b	Indumentum of peltate and stellate scales (or with at least some of the scales with a long fimbriate margin)	**60**
26a (25)	Scales densely covering lower surface of leaflet	**27**
26b	Scales ± absent to numerous on lower surface of leaflet	**31**
31a (26)	Scales few to numerous on lower surface of leaflet	**32**
31b	Scales ± absent from lower surface of leaflet but may densely cover the midrib below and immediately adjacent to it and occasionally on the lateral veins	**46**
46a (31)	Scales densely covering the midrib on lower surface of leaflet and immediately adjacent to the midrib, occasionally also on the lateral veins	**47**
46b	Scales ± absent from lower surface of leaflet	**54**
47a (46)	Scales large (many 0.2 mm across), orange-brown, reddish-brown or almost white, with a tendency to flake off	**48**
47b	Scales less than 0.2 mm across or if larger, then dark reddish-brown or purplish-brown and adhering closely to the leaflet	**50**
50a (47)	Anthers and/or staminal tube with simple white hairs	**51**
50b	Anthers and staminal tube without hairs	**52**
52a (50)	Leaflets with purplish-brown fimbriate peltate scales densely covering the midrib below and ± absent from the rest of the lower surface of the leaflet	**34. *A. glabrata***
52b (50)	Leaflets with dark reddish-brown peltate scales numerous on the midrib below	**53**
53a (52)	Leaflets 7–23, stellate scales absent	**53a (i) & (ii)**
53a(i)	Leaflets 11–23, 50–88 cm long, 36–54 cm wide, fruits dehiscent, at least 12.5 cm long and 10 cm wide	***A. mackiana***
53a (ii)	Leaflets (7-)9–13(-15), 5–18.5 cm long, 1.5–4.5 cm wide, fruits indehiscent, 1.5–3 cm long, 2–3.5 cm wide	**33. *A. scortechinii***
53b	Leaflets 3–5(-7), some stellate scales interspersed among the peltate scales	**50. *A. odoratissima***
69a (24)	Leaflets with few to densely covered with stellate hairs or scales on the lower surface; when sparse, some hairs or scales occur evenly distributed between the veins and their presence visible with the naked eye	**70**
69b	Leaflets without or with few hairs on the lower surface, with scales visible only with a lens or densely covered with hairs on the midrib only, few and unevenly scattered on the rest of the lower surface	**88**
88a (69)	Lower surface of leaflet with numerous stellate or peltate scales	**89**
88b	Leaflets with hairs or scales few on the lower surface between the veins when mature, but sometimes densely covering the midrib	**93**
93a (88)	Stellate hairs or scales more than 0.15 mm in diam., numerous on or densely covering the midrib, sometimes also on the lateral veins, almost absent elsewhere	**94**
93b	Stellate hairs or scales either very small, less than 0.15 mm in diameter, or almost totally absent from the midrib below and from the rest of the lower surface of leaflet	**109**
94a (93)	Leaves ± sessile or with a short peduncle of not more than 1 cm; the basal part of leaflets much smaller than the rest and subrotund	**61. *A. subsessilis***
94b	Leaves not sessile, the basal leaflets only slightly smaller than the rest and of similar shape	**95**
95a (94)	Reticulation subprominent on lower surface and often on upper surface of leaflet	**96**
95b	Reticulation may be visible, but not subprominent	**97**
97a (96)	Petals 3, densely covered with stellate scales on the outside; fruits dehiscent	**9. *A. lepidopetala***
97b	Petals 5, without scales on the outside, fruits indehiscent	**98**
98a (97)	Tree unbranched; leaflets shiny	**69. *A. coriacea***
98b	Tree branched; leaflets not shiny	**99**
99a (98)	Fruit c. 0.5 cm in diameter, with few stellate scales	**64. *A. aherniana***
99b	Ripe fruit 1 cm or more in diameter, with dense indumentum	**100**
100a (99)	Leaflet apex with a parallel-sided acumen	**101**
100b	Leaflet apex with a tapering acumen	**102**
101a (100)	Leaflets coriaceous	**42. *A. forbesii***
101b	Leaflets not coriaceous	**101b (i) & (ii)**
101b(i)	lateral veins subprominent	**40. *A. leptantha***
101b(ii)	lateral veins not raised	**43a *A. nyaruensis***

### Markers and trees

**Figure 4.**
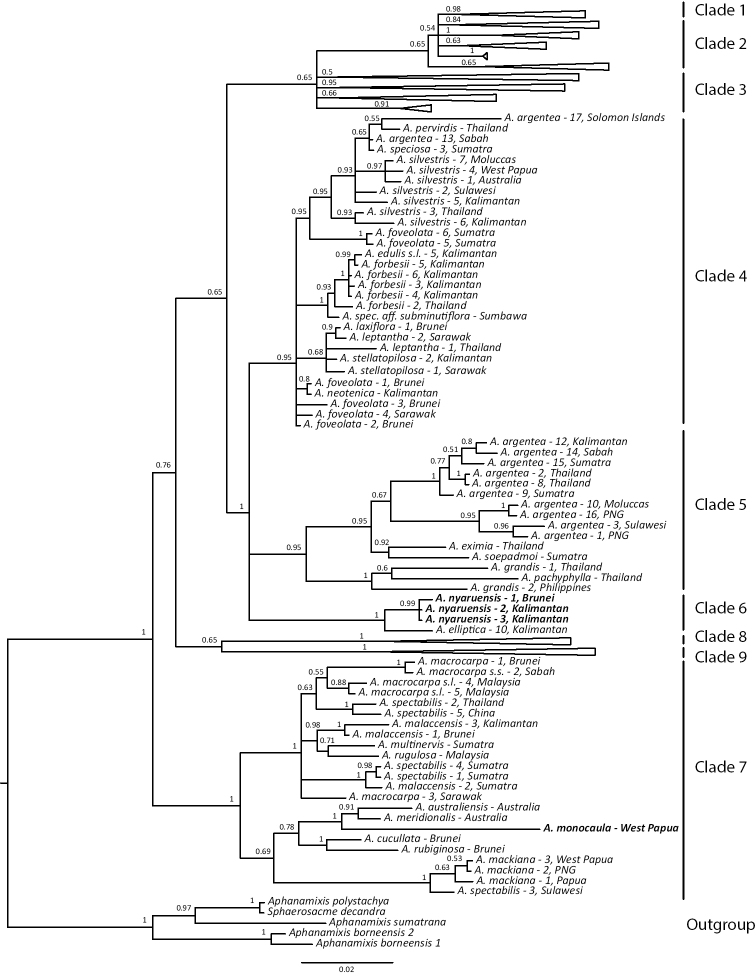
Majority-rule consensus tree of the combined nuclear dataset (ITS and ETS). Node values indicate Bayesian posterior probabilities. Clade numbers on the right refer to the clades identified by Grudinski et al. (2014). Clades outside the focus of this study were collapsed. Accessions of the new species (*A. nyaruensis* and *A. monocaula*) are highlighted in bold.

The final lengths of our alignments were 1006 bp (ITS), 515 bp (ETS), and 600 bp (*rps15-ycf1*). The results of our phylogenetic analyses of the combined nuclear data were largely congruent with the infrageneric relationships of Grudinski et al. (2014). Individual analysis of the plastid data, however, resulted in a largely unresolved tree (tree not shown). Furthermore, the combination of all markers led to an overall decrease of resolution and support as compared to the nuclear dataset (partly due to the high degree of missing data in the *rps15-ycf1* data). Given that we did not find any strongly supported disagreements between the plastid and nuclear data, we here present only the results of the analyses of the combined nuclear (ITS and ETS) dataset (Fig. 4). Both new species were found to be phylogenetically well supported. *Aglaia monocaula* was found to be closely related to other members of section Amoora (*A. meridionalis*, *A. australiensis*) from Australia. All samples of *A. nyaruensis* formed a strongly supported clade (pp = 0.99), with an accession of *A. elliptica* from Kalimantan as sister species. Both species belong to section Aglaia and have an indumentum of stellate hairs and scales.

**DNA barcodes**

Three-locus DNA barcodes (Table 1) are provided as aids for the identification of the two new species of *Aglaia*. On purpose, we did not use the 2-locus combination of *rbcL* and *matK* as originally recommended by the CBOL Plant Working Group (Hollingsworth et al. 2009a) or other previously recommended chloroplast markers, as previous phylogenetic and barcoding studies provided evidence for their insufficient taxonomic resolution at species level in the Meliaceae (e.g., Muellner et al. 2003, Muellner et al. 2011). ITS was proposed by Kress et al. (2005) as potential barcoding region, and has repeatedly been suggested as additional marker in case resolution with plastid markers was not sufficient in the group under investigation (Chase et al. 2005; Hollingsworth et al. 2009b; also compare the review by Shneyer 2009; and many others since then). In previous phylogenetic studies of *Aglaia*, ITS has so far been shown to constitute the most informative DNA region out of all markers investigated (e.g., Muellner et al. 2005, Grudinski et al. 2014). Recently, Dong et al. (2012) and Dong et al. (2015) found that parts of the plastid gene *ycf1* were very variable across flowering plants, indicating that this marker might be a useful barcode region. Finally, tests across several Meliaceae genera indicated that ETS might be another useful, i.e. informative, marker.

**Table 1.** Voucher information and GenBank accession numbers for *Aglaia monocaula* and *A. nyaruensis*.

Taxon	Locality	Voucher	ITS	ETS	*rps15-ycf1*
*A. monocaula*	West Papua	Polak 1221 (FHO)	MT439806	MT439713	MT409504
*A. nyaruensis*	Kalimantan	Sidiyasa et al. 1422 (L)	MT439808	MT439716	MT409506
	Kalimantan	G. Laman et al. 1397 (A)	MT439807	MT439715	MT409505
	Brunei	Muellner et al. 2039 (K,BRUN)	KF212126	MT439714	–

## Supplementary Material

XML Treatment for Aglaia monocaula

XML Treatment for Aglaia nyaruensis

XML Treatment for Aglaia mackiana

## Supplementary materials

Supplementary material 1 Table S1. Voucher information, origin, and GenBank accession numbers for all species included in this study Data type molecular data Explanation note New GenBank accessions (this study) are highlighted in bold. https://binary.pensoft.net/file/440312This dataset is made available under the Open Database License (http://opendatacommons.org/licenses/odbl/1.0/). The Open Database License (ODbL) is a license agreement intended to allow users to freely share, modify, and use this Dataset while maintaining this same freedom for others, provided that the original source and author(s) are credited.Caroline M. Pannell, Jan Schnitzler, Alexandra N. Muellner-Riehl

## References

[B1] Baldwin BG Markos S (1998) Phylogenetic Utility of the External Transcribed Spacer (ETS) of 18S-26S rDNA: Congruence of ETS and ITS Trees of *Calycadenia* (Compositae). Molecular Phylogenetics and Evolution 10(3): 449–463. 10.1006/mpev.1998.0545 10051397

[B2] Chase MW Salamin N Wilkinson M Dunwell JM Kesanakurthi RP Haidar N Savolainen V (2005) Land plants and DNA barcodes: Short-term and long-term goals. Philosophical Transactions of the Royal Society of London. Series B, Biological Sciences 360(1462): 1889–1895. 10.1098/rstb.2005.1720 PMC1609218 16214746

[B3] Clarkson JJ Pennington TD Chase MW Haynes G Engstrand R Kaye M Michalak I Muellner-Riehl AN (2016) Phylogenetic relationships in *Trichilia* (Meliaceae) based on ribosomal ITS sequences. Phytotaxa 259(1): 6–17. 10.11646/phytotaxa.259.1.4

[B4] Darriba D Taboada GL Doallo R Posada D (2012) jModelTest 2: More models, new heuristics and parallel computing. Nature Methods 9(8): 772 10.1038/nmeth.2109 PMC4594756 22847109

[B5] Dong W Liu J Yu J Wang L Zhou S (2012) Highly Variable Chloroplast Markers for Evaluating Plant Phylogeny at Low Taxonomic Levels and for DNA Barcoding. PLoS One 7(4): e35071. 10.1371/journal.pone.0035071 PMC3325284 22511980

[B6] Dong W Xu C Li C Sun J Zuo Y Shi S Cheng T Guo J Zhou S (2015) ycf1, the most promising plastid DNA barcode of land plants. Scientific Reports 5(1): 8348 10.1038/srep08348 25672218 PMC4325322

[B7] Edgar RC (2004) MUSCLE: Multiple sequence alignment with high accuracy and high throughput. Nucleic Acids Research 32(5): 1792–1797. 10.1093/nar/gkh340 15034147 PMC390337

[B8] Gama RL Muellner-Riehl AN Demarco D Pirani JR (2020) Evolution of reproductive traits in the mahagony family (Meliaceae). Journal of Systematics and Evolution.

[B9] Grudinski M Pannell CM Chase MW Ahmad JA Muellner-Riehl AN (2014) An evaluation of taxonomic concepts of the widespread plant genus *Aglaia* and its allies across Wallace’s Line (tribe Aglaieae, Meliaceae). Molecular Phylogenetics and Evolution 73: 65–76. 10.1016/j.ympev.2014.01.025 24495856

[B10] Hershkovitz MA Zimmer EA (1996) Conservation patterns in angiosperm rDNA ITS2 sequences. Nucleic Acids Research 24(15): 2857–2867. 10.1093/nar/24.15.2857 8760866 PMC146035

[B11] Hollingsworth PM Forrest LL Spouge JL Hajibabaei M Ratnasingham S van der Bank M Chase MW Cowan RS Erickson DL Fazekas AJ Graham SW James KE Kim K-J Kress WJ Schneider H van AlphenStahl J Barrett SCH van den Berg C Bogarin D Burgess KS Cameron KM Carine M Chacon J Clark A Clarkson JJ Conrad F Devey DS Ford CS Hedderson TAJ Hollingsworth ML Husband BC Kelly LJ Kesanakurti PR Kim JS Kim Y-D Lahaye R Lee H-L Long DG Madrinan S Maurin O Meusnier I Newmaster SG Park C-W Percy DM Petersen G Richardson JE Salazar GA Savolainen V Seberg O Wilkinson MJ Yi D-K Little DP (2009a) A DNA barcode for land plants. Proceedings of the National Academy of Sciences of the United States of America 106(31): 12794–12797. 10.1073/pnas.0905845106 19666622 PMC2722355

[B12] Hollingsworth ML Clark AA Forrest LL Richardson J Pennington RT Long DG Cowan R Chase MW Gaudeul M Hollingsworth PM (2009b) Selecting barcoding loci for plants: Evaluation of seven candidate loci with species-level sampling in three divergent groups of land plants. Molecular Ecology Resources 9(2): 439–457. 10.1111/j.1755-0998.2008.02439.x 21564673

[B13] Huelsenbeck JP Ronquist F (2001) MrBayes: Bayesian inference of phylogenetic trees. Bioinformatics (Oxford, England) 17(8): 754–755. 10.1093/bioinformatics/17.8.754 11524383

[B14] IUCN (2012) IUCN Red List Categories and Criteria - version 3.1, (2^nd^ edn). IUCN, Gland and Cambridge, 32 pp https://portals.iucn.org/library/node/10315

[B15] Jobes DV Thien LB (1997) A conserved motif in the 5.8S ribosomal RNA (rRNA) gene is a useful diagnostic marker for plant internal transcribed spacer (ITS) sequences. Plant Molecular Biology Reporter 15(4): 326–334. 10.1023/A:1007462330699

[B16] Kearse M Moir R Wilson A Stones-Havas S Cheung M Sturrock S Buxton S Cooper A Markovitz S Duran C Thierer T Ahton B Meintjes P Drummoned A (2012) Geneious Basic: An integrated and extendable desktop software platform for the organization and analysis of sequence data. Bioinformatics (Oxford, England) 28(12): 1647–1649. 10.1093/bioinformatics/bts199 PMC3371832 22543367

[B17] Köcke AV Muellner-Riehl AN Cáceres O Pennington TD (2015) *Cedrela ngobe* (Meliaceae), a new species from Panama and Costa Rica. Edinburgh Journal of Botany 72(2): 225–233. 10.1017/S0960428615000098

[B18] Kress WJ Wurdack KJ Zimmer EA Weigt LA Janzen DH (2005) Use of DNA barcodes to identify flowering plants. Proceedings of the National Academy of Sciences of the United States of America 102(23): 8369–8374. 10.1073/pnas.0503123102 15928076 PMC1142120

[B19] Liu J-S Schardl CL (1994) A conserved sequence in internal transcribed spacer 1 of plant nuclear rRNA genes. Plant Molecular Biology 26(2): 775–778. 10.1007/BF00013763 7948932

[B20] Mack A (1995a) Seed dispersal by the Dwarf Cassowary, *Casuarius bennettii* in Papua New Guinea. Ph.D. dissertation, University of Miami. https://www.researchgate.net/publication/34803848_Seed_dispersal_by_the_Dwarf_Cassowary_Casuarius_bennetti_in_Papua_New_Guinea#fullTextFileContent

[B21] Mack A (1995b) Distance and non-randomness of dispersal by the Dwarf Cassowary, *Casuarius bennettii*. Ecography 18(3): 286–295. 10.1111/j.1600-0587.1995.tb00131.x

[B22] Muellner AN Mabberley DJ (2008) Phylogenetic position and taxonomic disposition of *Turraea breviflora* Ridl. (Meliaceae), a hitherto enigmatic species. Blumea 53(3): 607–616. 10.3767/000651908X607549

[B23] Muellner AN Samuel R Johnson SA Cheek M Pennington TD Chase MW (2003) Molecular phylogenetics of Meliaceae (Sapindales) based on nuclear and plastid DNA sequences. American Journal of Botany 90(3): 471–480. 10.3732/ajb.90.3.471 21659140

[B24] Muellner AN Samuel R Chase MW Pannell CM Greger H (2005) *Aglaia* (Meliaceae): An evaluation of taxonomic concepts based on DNA data and secondary metabolites. American Journal of Botany 92(3): 534–543. 10.3732/ajb.92.3.534 21652432

[B25] Muellner AN Samuel R Chase MW Coleman A Stuessy TF (2008a) An evaluation of tribes and of generic relationships in Melioideae (Meliaceae) based on nuclear ITS ribosomal DNA. Taxon 57(1): 98–106.

[B26] Muellner AN Pannell CM Coleman A Chase MW (2008b) The origin and evolution of Indomalesian, Australasian and Pacific island biotas: Insights from *Aglaieae* (Meliaceae, Sapindales). Journal of Biogeography 35(10): 1769–1789. 10.1111/j.1365-2699.2008.01935.x

[B27] Muellner AN Pannell CM Greger H (2009a) Genetic diversity and geographic structure in *Aglaia elaeagnoidea* (Meliaceae, Sapindales), a morphologically complex tree species, near the two extremes of its distribution. Blumea 54(1–3): 207–216. 10.3767/000651909X476175

[B28] Muellner AN Pennington TD Chase MW (2009b) Molecular phylogenetics of Neotropical Cedreleae (mahogany family, Meliaceae) based on nuclear and plastid DNA sequences reveal multiple origins of “*Cedrela odorata*”. Molecular Phylogenetics and Evolution 52(2): 461–469. 10.1016/j.ympev.2009.03.025 19348956

[B29] Muellner AN Schaefer H Lahaye R (2011) Evaluation of DNA barcodes for economically important timber species of the mahogany family (Meliaceae). Molecular Ecology Resources 11(3): 450–460. 10.1111/j.1755-0998.2011.02984.x 21481203

[B30] Müller C Schulte FW Lange-Grünweller K Obermann W Madhugiri R Pleschka S Zieburh J Hartmann R Grünweller A (2018) Broad-spectrum antiviral activity of the eIF4A inhibitor silvestrol against corona- and picornaviruses. Antiviral Research 150: 123–129. 10.1016/j.antiviral.2017.12.010 29258862 PMC7113723

[B31] Müller C Obermann W Schulte FW Lange-Grünweller K Oestereich L Elgner F Glitscher M Hildt E Singh K Wendel HG Hartmann RK Ziebuhr J Grünweller A (2020) Comparison of broad-spectrum antiviral activities of the synthetic rocaglate CR-31-B (−) and the eIF4A-inhibitor Silvestrol. Antiviral Research 175: 104706. 10.1016/j.antiviral.2020.104706 PMC7114339 31931103

[B32] Pannell CM (1992) A taxonomic monograph of the genus *Aglaia* Lour. (Meliaceae). Kew Bulletin Additional Series XVI. HMSO, London, United Kingdom, viii + 1–379.

[B33] Pannell CM (1995) *Aglaia* In: Mabberley DJ, Pannell CM, Sing AM (Authors), Meliaceae Flora Malesiana I(12): 194–314. Foundation Flora Malesiana, Leiden, Netherlands. https://www.semanticscholar.org/paper/Flora-Malesiana%3A-Series-I.-Spermatophyta-Volume-12%2C-Mabberley-Pannell/f7bfe88a821e26ccb1289d502b9988b7a3011490

[B34] Pannell CM (1997) A new, cassowary-dispersed, species of *Aglaia* (Meliaceae, section Amoora) from Papua New Guinea. Kew Bulletin 52(3): 715–717. 10.2307/4110302

[B35] Pannell CM (1998a) Taxonomy, ecology and reproductive biology of *Aglaia* (Meliaceae). In: Hopkins HCF Huxley CR Pannell CM Prance GT White F (Eds) The biological monograph: The importance of field studies and functional syndromes for taxonomy and evolution of tropical plants, A Festschrift for Frank White.Royal Botanic Gardens Kew, London, United Kingdom, 59–77. https://link.springer.com/article/10.2307/2666614

[B36] Pannell CM (1998b) Species delimitation in *Aglaia*. In: Hopkins HCF Huxley CR Pannell CM Prance GT White F (Eds) The biological monograph, The importance of field studies and functional syndromes for taxonomy and evolution of tropical plants, A Festschrift for Frank White.Royal Botanic Gardens Kew, London, United Kingdom, 124–127. https://link.springer.com/article/10.2307/2666614

[B37] Pannell CM (2004) Three new species, two new subspecies and five new combinations at the subspecific level in *Aglaia* Lour. (Meliaceae). Kew Bulletin 59(1): 87–94. 10.2307/4111078

[B38] Pannell CM (2007) *Aglaia* (Meliaceae). In: Soepadmo E, et al (Eds) Tree Flora of Sabah and Sarawak 6: 24–107. https://www.nhbs.com/3/series/tree-flora-of-sabah-and-sarawak?qtview=154602

[B39] Pannell CM (2019) *Aglaia mabberleyi* Pannell (Meliaceae), a new species from Borneo. Gardens’ Bulletin (Singapore) 71(Suppl. 2): 189–195. 10.26492/gbs71(suppl.2).2019-02

[B40] Pennington TD Muellner AN (2010) A monograph of *Cedrela* DH Books, Sherborne, UK, 1–112. https://www.nhbs.com/a-monograph-of-cedrela-book

[B41] Prince LM (2015) Plastid primers for angiosperm phylogenetics and phylogeography. Applications in Plant Sciences 3(6): 1400085 10.3732/apps.1400085 PMC4467757 26082876

[B42] Rambaut A Drummond AJ Xie D Baele G Suchard MA (2018) Posterior summarization in Bayesian phylogenetics using Tracer 1.7. Systematic Biology 67(5): 901–904. 10.1093/sysbio/syy032 29718447 PMC6101584

[B43] Ronquist F Huelsenbeck JP (2003) MRBAYES 3: Bayesian phylogenetic inference under mixed models. Bioinformatics (Oxford, England) 19(12): 1572–1574. 10.1093/bioinformatics/btg180 12912839

[B44] Shneyer VS (2009) DNA barcoding is a new approach in comparative genomics of plants. Russian Journal of Genetics 45(11): 1267–1278. 10.1134/S1022795409110027 20058792

