# Supplementary material for: Two new species and a new species record of Aglaia (Meliaceae) from Indonesia
Source: PhytoKeys. 2020 Aug 7;155:33–51. doi: 10.3897/phytokeys.155.53833 (PMC7428463; doi:10.3897/phytokeys.155.53833)
Supplement: Supplementary material 1 — Table S1. Voucher information, origin, and GenBank accession numbers for all species included in this study [file phytokeys-155-033-s001.pdf]

Table S1. Voucher information, origin, and GenBank accession numbers for all species included in this study. New GenBank accessions (this study) are highlighted in bold.

Species	#	Collector number and location of herbarium voucher	Origin	ITS	rps15	ETS
A. agglomerata Merr. & Perry	1	Pennington 8063 (FHO)	New Guinea	KF212014	-	-
A. agglomerata Merr. & Perry	2	Schodde (and Craven) 4357 (US)	New Guinea	KF212015	-	-
A. agglomerata Merr. & Perry	3	Takeuchi and Arna 16362 (US)	New Guinea	KF212016	-	-
A. agglomerata Merr. & Perry		Takeuchi et al. 13685 (US)	New Guinea	KF212099	-	-
A. aherniana Perkins		Reynoso and Fuentes 24626 (K)	Philippines	KF212017	-	-
A. amplexicaulis A.C.Sm.		Kururshi 13884 (K)	Fiji	KF212018	-	-
A. angustifolia (Miq.) Miq.		Muellner et al. 2033 (K, BRUN)	Brunei	KF212019	-	-
A. apiocarpa Hiern.		Mabberley and Balasubramaniam 2432 (FHO)	Sri Lanka	KF212021	-	-
A. archboldiana A.C.Smith		Greger HG696 (WU)	Fiji	AY695524	-	**MT439653**
A. argentea Blume	1	Takeuchi et al. 13071 (K)	Papua New Guinea	KF212022	-	-
A. argentea Blume	2	Greger HG532 (WU)	Thailand	AY695525	-	-
A. argentea Blume	3	Coode 6212 (L)	Sulawesi	KF212023	-	-
A. argentea Blume	4	E.F. de Vogel 5927 (L)	Sulawesi	-	**MT409465**	-
A. argentea Blume	5	E.F. de Vogel & J.J. Vermeulen 6562 (L)	Sulawesi	-	**MT409466**	**MT439655**
A. argentea Blume	6	E.F. de Vogel 5532 (L)	Sulawesi	-	**MT409467**	-
A. argentea Blume	7	D.D. Soejarto & D.A. Madulid 6824 (L)	Palawan	-	**MT409468**	**MT439656**
A. argentea Blume	8	H. Greger (WU, HBV)	Thailand	**MT439752**	**MT409469**	**MT439657**
A. argentea Blume	9	Krukoff, B. A. 4149 (US)	Sumatra	**MT439756**	**MT409470**	**MT439661**
A. argentea Blume	10	Robinson, C. B. 1990 (US)	Moluccas	**MT439758**	**MT409471**	**MT439662**
A. argentea Blume	11	T.J.F. Bangun, M.C. Merello; I.A. Rachman; B. Sau; R. Mahroji & I. Haris 26 (BO,L,MO)	Moluccas	-	-	**MT439654**
A. argentea Blume	12	A.C. Church, U.W. Mahyar, Afriastini 1908 (A)	Kalimantan	**MT439753**	-	**MT439658**
A. argentea Blume	13	Kanis & Kuripin ! (US)	Sabah	**MT439754**	-	**MT439659**
A. argentea Blume	14	SAN 53128 (US)	Sabah	**MT439755**	-	**MT439660**
A. argentea Blume	15	de Wilde 18718 (US)	Sumatra	**MT439757**	-	-
A. argentea Blume	16	Streimann, H. NGF 44029 (US)	Papua New Guinea	**MT439759**	-	-
A. argentea Blume	17	Tenoa, R. BISP 6214 (US)	Solomon Islands	**MT439760**	-	**MT439663**
A. australiensis Pannell		Greger HG662 (WU)	Australia	AY695571	-	-
A. basiphylla A. Gray	1	Greger HG692 (WU)	Fiji	AY695527	-	-
A. basiphylla A. Gray	2	Smith 8713 (L)	Fiji	KF212024	-	-
A. beccarii C.DC.		Muellner et al. 2024 (K, BRUN)	Brunei	KF212160	-	-
A. brassii Merr. & Perry		Frodin et al. 3121 (K)	New Guinea	KF212026	-	-
A. brownii Pannell		Symon 7793 (FHO)	Australia	KF212027	-	-
A. ceramica (Miquel) Pannell		de Vriese and Teijsmann s.n. (L)	Moluccas	KF212028	-	-
A. chittagonga Miq.		Greger HG756 (WU)	Bangladesh	AY695528	-	-

A. cooperae Pannell	1	Cooper et al. 02029 (FHO)	Australia	KF212031	-	-
A. cooperae Pannell	2	Hyland 10296 (FHO)	Australia	KF212032	-	-
A. coriacea Korth. ex Miq.	1	Muellner et al. 2032 (K, BRUN)	Brunei	EF491263	-	-
A. coriacea Korth. ex Miq.	2	H. Greger HG822 (WU, HBV)	Thailand	**MT439761**	**MT409472**	**MT439666**
A. coriacea Korth. ex Miq.	3	H. Greger HG822 (WU, HBV)	Thailand	**MT439762**	**MT409473**	**MT439667**
A. costata Merrill		Madulid et al. 1021 (L)	Philippines	**MT439763**	-	-
A. crassinervia Kurz ex Hiern		Greger and Vajirodya HG523 (WU)	Thailand	AY695530	-	-
A. cremea Merr. & Perry		Coode 8129 (K)	New Guinea	KF212034	-	**MT439665**
A. cucullata (Roxb.) Pellegrin		Brunei Museum staff s.n. (K)	Brunei	AY695572	-	-
A. cumingiana Turcz.		Beaman 9997 (FHO)	Sabah	KF212035	-	-
A. edulis (Roxb.) Wall.	1	Greger and Vajirodya HG905 (WU)	Thailand	AY695534	-	-
A. edulis (Roxb.) Wall.	2	van Balgooy 6899 (L)	Moluccas	KF212037	-	-
A. edulis (Roxb.) Wall.	3	Greger HG561 (WU)	Thailand	AY695550	-	-
A. edulis (Roxb.) Wall.	4	M.J.E. Coode 6244A (L)	Sulawesi	-	-	**MT439668**
A. edulis (Roxb.) Wall.	5	J.S. Burley, Tukirin et al 3033 (A)	Kalimantan	**MT439764**	-	**MT439669**
A. elaeagnoidea (A.Juss.) Benth.	1	Greger HG646 (WU)	Australia	EU340982	-	-
A. elaeagnoidea (A.Juss.) Benth.	2	Delray 9673 (FHO)	New Caledonia	KF212038	-	-
A. elaeagnoidea (A.Juss.) Benth.	3	Mabberley 2012 (FHO)	New Caledonia	KF212039	-	-
A. elaeagnoidea (A.Juss.) Benth.	4	Pacher HG922 (FHO)	Sri Lanka	EU310243	-	-
A. elaeagnoidea (A.Juss.) Benth.	5	Ambriansyah et al. AA2634 (K)	Sulawesi	KF212040	-	-
A. elliptica Blume	1	Greger MEL36 (WU)	Indonesia	AY695540	-	-
A. elliptica Blume	2	Podzorski SMHI873 (FHO)	Philippines	KF212043	-	-
A. elliptica Blume	3	Ambriansyah et al. AA2640 (K, L)	Sulawesi	KF212044	-	**MT439670**
A. elliptica Blume	4	Middleton et al. 898 (K)	Thailand	KF212045	-	-
A. elliptica Blume	6	A.C. Church, with U.W. Mahyar, A. Ruskandi & Nurdin 227 (A)	Kalimantan	**MT439765**	**MT409474**	**MT439672**
A. elliptica Blume	7	A.C. Church, with U.W. Mahyar, A. Ruskandi & Nurdin 213 (A)	Kalimantan	**MT439766**	**MT409475**	**MT439673**
A. elliptica Blume	8	A.C. Church, with U.W. Mahyar, A. Ruskandi & Nurdin 504 (A)	Kalimantan	**MT439768**	**MT409476**	**MT439674**
A. elliptica Blume	9	E.F. de Vogel & J.J. Vermeulen 6993 (L)	Sulawesi	-	-	**MT439671**
A. elliptica Blume	10	A.C. Church, with U.W. Mahyar, A. Ruskandi & Nurdin 687 (A)	Kalimantan	**MT439767**	-	-
A. elliptica Blume *ssp. elliptica*		Pannell 1052 (FHO)	Peninsular Malaysia	KF212047	-	-
A. euryanthera Harms	1	M. Sands 6627 (L)	West Papua	-	**MT409477**	-
A. euryanthera Harms	2	M. Kato et al. C-5201 (A)	Moluccas	**MT439769**	**MT409478**	**MT439675**
A. evansensis A.C.Sm.	1	Smith 4080 (US)	Fiji	KF212049	-	-
A. evansensis A.C.Sm.	2	Smith 4152 (K)	Fiji	KF212050	-	**MT439676**
A. eximia Miq		Greger and Vajirodya HG817 (WU)	Thailand	AY695542	-	**MT439677**
A. exstipulata (Griffith) Theobald	1	Pannell 1389 (FHO)	Peninsular Malaysia	EU310250	-	-
A. exstipulata (Griffith) Theobald	2	Greger and Vajirodya HG819 (WU)	Thailand	AY695544	-	-
A. ferruginea C.T. White & W.D. Francis	1	Greger HG665 (WU)	Australia	AY695565	-	-
A. ferruginea C.T. White & W.D. Francis	2	Fell and Stanton 9275 (FHO)	Australia	KF212054	-	**MT439678**

A. ferruginea C.T. White & W.D. Francis	3	Gray 2896 (FHO)	Australia	EU310256	-	-
A. ferruginea C.T. White & W.D. Francis	4	Hyland 6621 (K)	Australia	EU310257	-	-
A. ferruginea C.T. White & W.D. Francis	5	Irvine 1641 (K)	Australia	EU310258	-	-
A. flavescens C.DC.		Takeuchi et al. 15179 (K)	New Guinea	KF212055	-	-
A. forbesii King	1	Pannell et al. 2568 (K, BRUN)	Brunei	KF212056	-	-
A. forbesii King	2	H. Greger HG530 (WU, HBV)	Thailand	**MT439770**	**MT409479**	**MT439679**
A. forbesii King	3	A.C. Church, U.W. Mahyar, Indah, Ismail & Hamzah 1020 (A)	Kalimantan	**MT439772**	**MT409480**	**MT439681**
A. forbesii King	4	A.C. Church, U.W. Mahyar, Indah, Ismail & Hamzah 1364 (A)	Kalimantan	**MT439773**	**MT409481**	**MT439682**
A. forbesii King	5	A.C. Church, U.W. Mahyar, Afriastini 1598 (A)	Kalimantan	**MT439774**	**MT409482**	**MT439683**
A. forbesii King	6	A.C. Church, with U.W. Mahyar, A. Ruskandi & Nurdin 621 (A)	Kalimantan	**MT439771**	-	**MT439680**
A. foveolata Pannell	1	Muellner et al. 2038 (K, BRUN)	Brunei	KF212058	-	-
A. foveolata Pannell	2	Muellner et al. 2045 (K, BRUN)	Brunei	KF212061	-	-
A. foveolata Pannell	3	Ariffin Kalat ARK 103 (A)	Brunei	-	**MT409483**	**MT439685**
A. foveolata Pannell	4	Banyeng, Nudong et al. S 25483 (A)	Sarawak	**MT439775**	-	-
A. foveolata Pannell	5	Burley et al. 1759 (US)	Sumatra	**MT439776**	-	**MT439686**
A. foveolata Pannell	6	J.S. Burley, Tukirin et al. 1357 (A)	Sumatra	-	-	**MT439684**
A. fragilis A.C.Sm.		Melvill 7044 (K)	Fiji	KF212062	-	-
A. glabrata Teijsm. & Binn.		Wilkie 93349 (K)	Kalimantan	AY695547	-	-
A. grandis Korth. in Miq.	1	Greger HG571 (WU)	Thailand	AY695548	-	-
A. grandis Korth. in Miq.	2	Soejarto and Madulid 6109 (L)	Philippines	KF212064	-	-
A. heterotricha A.C.Sm.		Parls 16305 (BM)	Tonga	KF212065	-	-
A. hiernii King		T.G. Laman, A. Ismail, Rachman, Edi Mirmanto TL1054 (A)	Kalimantan	**MT439777**	**MT409484**	**MT439687**
A. integrifolia Pannell		Mabberley 1777 (FHO)	New Guinea	KF212068	-	-
A. korthalsii Miq.	1	Muellner et al. 2041 (K, BRUN)	Brunei	EF491264	-	-
A. korthalsii Miq.	2	Johansson, Nybom and Riebe 205 (L)	Sulawesi	KF212069	-	-
A. korthalsii Miq.	3	Greger and Vajirodya HG807 (WU)	Thailand	AY695549	-	-
A. korthalsii Miq.	4	P.J.A. Kessler, Ramadhanil, B. Made & Muchlis PK 2959 (L)	Sulawesi	-	-	**MT439664**
A. lancifolia (Hook. f) Harms	1	Muellner et al. 2014 (K, BRUN)	Brunei	KF212070	-	-
A. lancifolia (Hook. f) Harms	2	A.C. Church, U.W. Mahyar, Afriastini 1496 (A)	Kalimantan	**MT439778**	**MT409485**	**MT439688**
A. lawii (Wight) C.J. Saldanha	1	Mauriasi et al. BSIP16172 (L)	Solomon Islands	KF212165	-	-
A. lawii (Wight) C.J. Saldanha	2	Greger HG488 (WU)	Thailand	AY695574	-	-
A. lawii (Wight) C.J. Saldanha	3	Pennington 8115 (FHO)	New Britain	KF212168	-	-
A. lawii (Wight) C.J. Saldanha	4	A.C. Church, with U.W. Mahyar, A. Ruskandin & Nurdin 136 (A)	Kalimantan	**MT439779**	**MT409486**	**MT439689**
A. lawii (Wight) C.J. Saldanha	5	A.C. Church, with U.W. Mahyar, A. Ruskandin & Nurdin 583 (A)	Kalimantan	**MT439780**	**MT409487**	**MT439690**
A. lawii (Wight) C.J. Saldanha	6	T.G. Laman, A. Ismail, Rachman, Edi Mirmanto TL299 (A)	Kalimantan	-	**MT409488**	-
A. lawii (Wight) C.J. Saldanha	7	T.G. Laman, A. Ismail, Rachman, Edi Mirmanto TL970 (A)	Kalimantan	**MT439781**	**MT409489**	**MT439691**
A. lawii (Wight) C.J. Saldanha	8	T.G. Laman, A. Ismail, Rachman, Edi Mirmanto TL1121 (A)	Kalimantan	**MT439782**	**MT409490**	**MT439692**
A. lawii (Wight) C.J. Saldanha	9	T.G. Laman, A. Ismail, Rachman, Edi Mirmanto TL1402 (A)	Kalimantan	**MT439783**	**MT409491**	**MT439693**
A. lawii (Wight) C.J. Saldanha	10	K. Armstrong et al. 1217 (NY)	Myanmar	**MT439785**	**MT409492**	**MT439695**

A. lawii (Wight) C.J. Saldanha	11	K. Armstrong et al. 1702 (NY)	Myanmar	**MT439786**	**MT409493**	**MT439696**
A. lawii (Wight) C.J. Saldanha	12	K. Armstrong et al. 2356 (NY)	Myanmar	**MT439787**	**MT409494**	**MT439697**
A. lawii (Wight) C.J. Saldanha	13	Wen, H.Q. w079 (US)	China	**MT439788**	**MT409495**	**MT439698**
A. lawii (Wight) C.J. Saldanha	14	Stocks et al. s.n. (NY)	India	**MT439784**	-	**MT439694**
A. lawii (Wight) C.J. Saldanha	15	C.J. Saldanha 16032 (US)	India	-	-	**MT439699**
A. lawii (Wight) C.J. Saldanha	16	Poilane, E. 6673 (US)	Vietnam	**MT439789**	-	**MT439700**
A. lawii (Wight) C.J. Saldanha	17	Rock, J. F. 1967 (US)	Myanmar	**MT439790**	-	**MT439701**
A. lawii (Wight) C.J. Saldanha	18	Hartley, T. G. TGH 10922 (US)	Papua New Guinea	**MT439791**	-	**MT439702**
A. lawii (Wright) C.J. Saldanha *ssp. oligocarpa* (Miq.) Pannell		Muellner et al. 2003 (K, BRUN)	Brunei	KF212169	-	-
A. laxiflora Miq.	1	Sands 5912 (BRUN)	Brunei	KF212072	-	-
A. laxiflora Miq.	2	Ambaiansyah AA 3046 (L)	Kalimantan	-	**MT409496**	-
A. lepiorrhachis Harms		Barker LAE66770 (K)	New Guinea	KF212073	-	-
A. leptantha Miq	1	Kerr 15476 (K)	Thailand	KF212074	-	-
A. leptantha Miq	2	Paie S27697 (K)	Sarawak	KF212075	-	-
A. leucophylla King	1	Wilkie 3364 (K)	Kalimantan	KF212077	-	-
A. leucophylla King	2	A.C. Church, U.W. Mahyar, Indah, Ismail & Hamzah 1022 (A)	Kalimantan	**MT439792**	**MT409497**	**MT439703**
A. leucophylla King	3	A.C. Church, U.W. Mahyar, Indah, Ismail & Hamzah 1300 (A)	Kalimantan	**MT439793**	**MT409498**	**MT439704**
A. leucophylla King	4	T.G. Laman, A. Ismail, Rachman, Edi Mirmanto TL997 (A)	Kalimantan	**MT439794**	-	**MT439705**
A. luzoniensis (Vidal) Merrill & Rolfe		Beaman 10600 (FHO)	Sabah	KF212078	-	-
A. mabberleyi Pannell	1	Lee et al. S.53843 (BRUN)	Sarawak	KF212104	-	-
A. mabberleyi Pannell	2	A.C. Church, U.W. Mahyar, Indah, Ismail & Hamzah 236 (A)	Kalimantan	**MT439795**	**MT409499**	**MT439706**
A. mabberleyi Pannell	3	A.C. Church, with U.W. Mahyar, A. Ruskandi & Nurdin 172 (A)	Kalimantan	**MT439796**	**MT409500**	**MT439707**
A. mabberleyi Pannell	4	A.C. Church, with U.W. Mahyar, A. Ruskandi & Nurdin 454 (A)	Kalimantan	**MT439797**	**MT409501**	**MT439708**
A. mackiana Pannell	1	McDonald and Ismail 3786 (K)	Papua	**MT439798**	-	-
A. mackiana Pannell	2	Mack 699 (FHO)	Papua New Guinea	**MT439799**	**MT409502**	**MT439710**
A. mackiana Pannell	3	Wanda Ave 4394 (L)	West Papua	-	-	**MT439709**
A. macrocarpa (Miq.) Pannell	1	Muellner et al. 2006 (K, BRUN)	Brunei	KF212146	-	-
A. macrocarpa (Miq.) Pannell	2	O. Toru SAN 92178 (L)	Sabah	**MT439800**	-	-
A. macrocarpa (Miq.) Pannell	3	P.S. Ashton S 19078 (L)	Sarawak	**MT439801**	-	-
A. macrocarpa (Miq.) Pannell	4	K.M. Kochumen FRI 16593 (L)	Peninsular Malaysia	**MT439802**	-	-
A. macrocarpa (Miq.) Pannell	5	K.M. Kochumen FRI 2219 (L)	Peninsular Malaysia	**MT439803**	-	-
A. macrostigma King		Pannell 1017 (FHO)	Peninsular Malaysia	KF212079	-	-
A. malabarica Sasidharan		Sasidharan 5657 (FHO)	India	KF212080	-	-
A. malaccensis (Ridley) J.A.R. Anderson	1	Muellner et al. 2035 (K, BRUN)	Brunei	KF212151	-	-
A. malaccensis (Ridley) J.A.R. Anderson	2	J.S. Burley, Tukirin et al. 1306 (L)	Sumatra	**MT439804**	**MT409503**	-
A. malaccensis (Ridley) J.A.R. Anderson	3	T.G. Laman, A. Ismail, Rachman, Edi Mirmanto TL1217 (A)	Kalimantan	**MT439805**	-	**MT439711**
A. mariannensis Merrill	1	Takamatsu 716 (FHO)	Caroline Islands	KF212081	-	**MT439712**
A. mariannensis Merrill	2	Taitano, R. 41 (L)	Mariana Islands	KF212082	-	-
A. mariannensis Merrill	3	Fosberg, F.R. 35492 (L)	Mariana Islands	KF212083	-	-

A. mariannensis Merrill	4	Stone and Krizman 15993 (E)	Mariana Islands	KF212084	-	-
A. meliosmoides Craib	1	Muellner et al. 2019 (K, BRUN)	Brunei	KF212085	-	-
A. meliosmoides Craib	2	Sinclair 10377 (US)	Sarawak	KF212087	-	-
A. meliosmoides Craib	3	Fosberg 43825 (US)	Sarawak	KF212121	-	-
A. membranifolia King	4	Pannell 1939 (FHO)	Sumatra	KF212088	-	-
A. meridionalis Pannell		Greger MEL22 (WU)	Australia	AY695577	-	-
A. monocaula Pannell		A.M. Polak 1221 (FHO)	West Papua	**MT439806**	**MT409504**	**MT439713**
A. multinervis Pannell		Pannell 1901 (FHO)	Sumatra	KF212152	-	-
A. neotenica Kostermans		Yii et al. S.51254 (BRUN)	Borneo	KF212090	-	-
A. nyaruensis Pannell	1	Muellner et al. 2039 (K, BRUN)	Brunei	KF212126	-	**MT439714**
A. nyaruensis Pannell	2	T.G. Laman, A. Ismail, Rachman, Edi Mirmanto TL1397 (A)	Kalimantan	**MT439807**	**MT409505**	**MT439715**
A. nyaruensis Pannell	3	K. Sidiyassa 1422 (A)	Kalimantan	**MT439808**	**MT409506**	**MT439716**
A. odorata Lour.		Greger and Vajirodya HG516 (WU)	Thailand	AY695551	-	-
A. odoratissima Blume	1	Muellner et al. 2018 (K, BRUN)	Brunei	KF212091	-	-
A. odoratissima Blume	2	Greger HG623 (WU)	Thailand	AY695553	-	-
A. odoratissima Blume	3	R. Hendrian, M.F. Newman, S. Scott, M. Nazre Saleh & Dadi Supriadi 980 (BO)	Sulawesi	-	-	**MT439717**
A. odoratissima Blume	4	A.C. Church, U.W. Mahyar, Indah, Ismail & Hamzah 1198 (A)	Kalimantan	**MT439809**	-	**MT439718**
A. odoratissima Blume	5	T.G. Laman, A. Ismail, Rachman, Edi Mirmanto TL1270 (A)	Kalimantan	**MT439810**	-	**MT439719**
A. odoratissima Blume	6	T.G. Laman, A. Ismail, Rachman, Edi Mirmanto TL411 (A)	Kalimantan	**MT439811**	-	**MT439720**
A. oligophylla Miq.	1	Burley et al. 1973 (K)	Sumatra	KF212092	-	-
A. oligophylla Miq.	2	Greger HG706 (WU)	Thailand	AY695554	-	-
A. oligophylla Miq.	3	A.C. Church, Ismail, A. Ruskandi 2401 (A)	Kalimantan	**MT439812**	**MT409507**	**MT439721**
A. pachyphylla Miq.		Greger and Vajirodya HG812 (WU)	Thailand	AY695555	-	-
A. parviflora C.DC.		van Balgooy 6731 (K)	Moluccas	KF212095	-	-
A. pervirdis Hiern		Greger HG13 (WU)	Thailand	AY695556	-	-
A. puberulanthera C.DC.	1	Takeuchi, W.N. et al. 13321 (L)	New Guinea	KF212097	-	-
A. puberulanthera C.DC.	2	Takeuchi et al. 23300 (FHO)	New Guinea	KF212098	-	-
A. rimosa (Blanco) Merr.	2	Cam Webb mx-885 (A)	Sulawesi	**MT439813**	-	-
A. rimosa (Blanco) Merr.	3	Frodin, D. G. NGF 26914 (US)	New Britain	-	-	**MT439723**
A. rimosa (Blanco) Merr.	4	Adduru, M. !5 (US)	Philippines	**MT439815**	-	**MT439724**
A. rimosa (Blanco) Merr.	5	Cenabre et al. For. Bur. 28521 (US)	Philippines	**MT439816**	-	**MT439725**
A. rimosa (Blanco) Merr.	6	Ramos, M. Bur. Sci 24112 (US)	Philippines	**MT439817**	-	**MT439726**
A. rimosa (Blanco) Merril	1	Utteridge 317 (FHO, A)	New Guinea	**MT439814**	**MT409508**	**MT439722**
A. rubiginosa (Hiern) Pannell		Suzuki K12994 (BRUN)	Brunei	KF212155	-	-
A. rubrivenia Merr. & Perry		BSIP 16992 (K)	Solomon Islands	KF212103	-	-
A. rufinervis (Blume) Bentv.	1	Pannell 1913 (FHO)	Sumatra	KF212107	-	-
A. rufinervis (Blume) Bentv.	2	T.G. Laman, A. Ismail, Rachman, Edi Mirmanto TL150 (A)	Kalimantan	**MT439818**	**MT409509**	**MT439727**
A. rugulosa Pannell		Mat Asri Kep. Field No. FRI 25591 (K)	Peninsular Malaysia	AY695578	-	-
A. saltatorum A.C.Sm.		Whistler 6768 (K)	Tonga	KF212109	-	**MT439728**

A. samoensis A. Gray	4	NT 11130 (L)	West Papua	-	**MT409510**	-
A. samoensis A.Gray	1	Greger HG752 (WU)	Samoa	AY695557	-	-
A. samoensis A.Gray	2	Pillon et al. 991 (NOU)	Wallis	KF212113	-	-
A. samoensis A.Gray	3	Pillon et al. 905 (NOU)	Wallis	KF212114	-	-
A. sapindina (F. Muell.) Harms	1	Greger HG669 (WU)	Australia	AY695558	-	-
A. sapindina (F. Muell.) Harms	2	H. Greger HG668 (WU, HBV)	Thailand	**MT439819**	**MT409511**	**MT439729**
A. scortechinii King		Mabberley 1634 (FHO)	Borneo	KF212115	-	-
A. sexipetala Griffith	1	Ambriansyah 1511 (K)	Kalimantan	AY695559	-	-
A. sexipetala Griffith	2	Siga and Towati 14911 (K)	New Guinea	KF212116	-	-
A. sexipetala Griffith	3	Cheng FRI23365 (K)	Peninsular Malaysia	KF212117	-	-
A. sexipetala Griffith	4	Greger and Vajirodya HG728 (WU)	Thailand	AY695532	-	-
A. sexipetala Griffith	5	A.C. Church, with U.W. Mahyar, A. Ruskandi & Nurdin 143 (A)	Kalimantan	-	-	**MT439730**
A. sexipetala Griffith	6	A.C. Church, with U.W. Mahyar, A. Ruskandi & Nurdin 629 (A)	Kalimantan	**MT439820**	**MT409512**	**MT439731**
A. silvestris (M. Roem.) Merr.	1	Cooper and Jensen 02041 (FHO)	Australia	KF212118	-	-
A. silvestris (M. Roem.) Merr.	2	Walker and Cahill DB108 (K)	Sulawesi	KF212119	-	-
A. silvestris (M. Roem.) Merr.	3	Greger HG719 (WU)	Thailand	**MT439821**	-	-
A. silvestris (M. Roem.) Merr.	4	A.M. Polak 1180 (L)	West Papua	-	**MT409513**	**MT439733**
A. silvestris (M. Roem.) Merr.	5	A.C. Church, U.W. Mahyar, Indah, Ismail & Hamzah 1263 (A)	Kalimantan	**MT439823**	**MT409514**	**MT439734**
A. silvestris (M. Roem.) Merr.	6	T.G. Laman, A. Ismail, Rachman, Edi Mirmanto TL295 (A)	Kalimantan	**MT439824**	**MT409515**	**MT439735**
A. silvestris (M. Roem.) Merr.	7	T.J.F. Bangun, L.D. Andriamahefarivo, R. Razakamalala, S.A. Lasut & A. Yani 391 (BO,L,MO)	Moluccas	**MT439822**	-	**MT439732**
A. simplicifolia (Bedd.) Harms	1	Greger HG484 (WU)	Thailand	**MT439826**	**MT409517**	**MT439737**
A. simplicifolia (Bedd.) Harms	2	H. Greger HG485 (WU, HBV)	Thailand	**MT439825**	**MT409516**	**MT439736**
A. soepadmoi Pannell		Laumonier s.n. (FHO)	Sumatra	KF212122	-	**MT439738**
A. spec. aff. saltatorum A.C.Sm.		Cabalion 2867 (K)	Vanuatu	KF212110	-	-
A. spec. aff. sessilifolia Pannell		H.P. Nooteboom 5822 (L)	Moluccas	-	**MT409464**	-
A. spec. aff. subminutiflora C. DC.		W.S. Hoover et al Deden 242 (A)	Sumbawa	**MT439751**	-	**MT439652**
A. spec. aff. tomentosa Teijsm. & Binn	1	McDonald and Ismail 3715 (K)	New Guinea	EU310253	-	-
A. spec. aff. tomentosa Teijsm. & Binn	2	Frodin et al. 2770 (K)	New Guinea	EU310254	-	-
A. spec. aff. tomentosa Teijsm. & Binn	3	Coode 6287 (K)	Sulawesi	EU310255	-	-
A. spec. nov. NG		Hartley TGH10476 (US)	New Guinea	KF212125	-	-
A. speciosa Blume	1	Herbarium H.D. Rijksen 201172 (L)	Sumatra	KF212128	-	-
A. speciosa Blume	2	Church et al. 2541 (K, A)	Kalimantan	**MT439827**	**MT409518**	**MT439739**
A. speciosa Blume	3	Krukoff, B. A. 4091 (US)	Sumatra	**MT439828**	-	**MT439740**
A. speciosa Blume	4	de Wilde 14817 (US)	Sumatra	**MT439829**	-	**MT439741**
A. spectabilis (Miq.) Jain & Bennet	1	Pannell 2083 Sheet 2 (FHO)	Sumatra	KF212159	-	-
A. spectabilis (Miq.) Jain & Bennet	2	Greger and Vajirodya HG864 (WU)	Thailand	AY695580	-	-
A. spectabilis (Miq.) Jain & Bennet	3	Neth.Ind.For.Service s.n. (L)	Sulawesi	KF212157	-	-
A. spectabilis (Miq.) Jain & Bennet	4	Krukoff, B. A. 4032 (US)	Sumatra	**MT439830**	-	**MT439742**
A. spectabilis (Miq.) Jain & Bennet	5	H.Y. Liang 63794 (US)	China	**MT439831**	-	**MT439743**

A. stellatopilosa Pannell	1	Paie S.37572 (FHO)	Sarawak	KF212130	-	**MT439745**
A. stellatopilosa Pannell	2	A.C. Church, with U.W. Mahyar, A. Ruskandin & Nurdin 458 (A)	Kalimantan	**MT439832**	**MT409519**	**MT439744**
A. subcuprea Merr. & Perry		Regalado 1563 (K)	New Guinea	KF212132	-	-
A. subminutiflora C.DC.	1	Conn 1747 (L)	New Guinea	KF212133	-	-
A. subminutiflora C.DC.	2	Takeuchi 11809 (K)	New Guinea	KF212134	-	-
A. subminutiflora C.DC.	3	Forster and Liddle PIF8677 (K)	Solomon Islands	KF212135	-	-
A. subsessilis Pannell		McDonald and Ismail 3609 (K)	Kalimantan	KF212136	-	-
A. tenuicaulis Hiern	1	Pannell 1207 Sheet 2 (FHO)	Peninsular Malaysia	KF212137	-	-
A. tenuicaulis Hiern	2	Greger HG901 (WU)	Thailand	AY695564	-	-
A. tenuicaulis Hiern *ssp. semengohensis* Pannell		Pennington 7952 (L)	Sarawak	KF212138	-	-
A. teysmanniana (Miq.) Miq.		Brader and Pacher HG635 (WU)	Thailand	AY695581	-	-
A. tomentosa Teijsm. & Binn	1	Greger HG543 (WU)	Thailand	AY695568	-	-
A. tomentosa Teijsm. & Binn	2	van Steenis 1101 (K)	Bunguran Island	**MT439833**	-	-
A. tomentosa Teijsm. & Binn	3	Arnold Arboretum IS425 (K)	Kalimantan	EU310251	-	-
A. tomentosa Teijsm. & Binn	4	Geesink et al. 7201 (K)	Thailand	EU310244	-	-
A. tomentosa Teijsm. & Binn	5	van Bensekom and Phengkhlai 703 (K)	Thailand	EU310245	-	**MT439750**
A. tomentosa Teijsm. & Binn	6	Asri FRI25502 (K)	Peninsular Malaysia	EU310246	-	**MT439749**
A. tomentosa Teijsm. & Binn	7	Soejarto and Fernando 7289 (K)	Philippines	EU310252	-	-
A. tomentosa Teijsm. & Binn	8	Sinclair and Edaño 9552 (K)	Philippines	KF212140	-	-
A. tomentosa Teijsm. & Binn	9	Madani SAN142621 (K)	Sabah	EU310248	-	-
A. tomentosa Teijsm. & Binn	10	Niyandhan et al. 297 (K)	Thailand	EF491265	-	-
A. tomentosa Teijsm. & Binn	11	Greger and Vajirodya HG698 (WU)	Thailand	AY695567	-	-
A. tomentosa Teijsm. & Binn	12	Greger and Vajirodya HG818 (WU)	Thailand	AY695566	-	-
A. tomentosa Teijsm. & Binn	15	T.G. Laman, A. Ismail, Rachman, Edi Mirmanto TL1240 (A)	Kalimantan	**MT439835**	-	**MT439747**
A. tomentosa Teijsm. & Binn.	13	A.C. Church, U.W. Mahyar, Indah, Ismail & Hamzah 1213 (A)	Kalimantan	**MT439834**	**MT409520**	**MT439746**
A. tomentosa Teijsm. & Binn.	14	A.C. Church, with U.W. Mahyar, A. Ruskandi & Nurdin 218 (A)	Kalimantan	**MT439836**	**MT409521**	**MT439748**
A. tomentosa Teijsm. & Binn. *ssp. cordata* (Hiern) Pannell	1	Muellner et al. 2040 (K, BRUN)	Brunei	EU310259	-	-
A. tomentosa Teijsm. & Binn. *ssp. cordata* (Hiern) Pannell	2	Burley et al. 396 (FHO)	Kalimantan	EU310260	-	-
A. tomentosa Teijsm. & Binn. *ssp. cordata* (Hiern) Pannell	3	Pannell 2036 (FHO)	Sumatra	EU310261	-	-
A. tomentosa Teijsm. & Binn. *ssp. cordata* (Hiern) Pannell	4	Pannell 2040 (FHO)	Sumatra	EU310262	-	-
A. tomentosa Teijsm. & Binn. *ssp. tomentosa*	1	Muellner et al. 2000 (K, BRUN)	Brunei	EU310263	-	-
A. tomentosa Teijsm. & Binn. *ssp. tomentosa*	2	Muellner et al. 2011 (K, BRUN)	Brunei	EU310264	-	-
A. tomentosa Teijsm. & Binn. *ssp. tomentosa*	3	Muellner et al. 2020 (K, BRUN)	Brunei	EU310265	-	-
A. tomentosa Teijsm. & Binn. *ssp. tomentosa*	4	Muellner et al. 2036 (K, BRUN)	Brunei	EU310267	-	-
A. tomentosa Teijsm. & Binn. *ssp. tomentosa*	5	Muellner et al. 2037 (K, BRUN)	Brunei	EU310266	-	-
A. tomentosa Teijsm. & Binn. *ssp. tomentosa*	6	Muellner et al. 2043 (K, BRUN)	Brunei	EU310249	-	-
A. unifolia P.T.Liet x M.Chen		Smith 5683 (L)	Fiji	KF212141	-	-

A. vitiensis A.C.Sm.	1	Greger HG691 (WU)	Fiji	AY695569	-	-
A. vitiensis A.C.Sm.	2	Smith 7762 (L)	Fiji	KF212143	-	-
A. vitiensis A.C.Sm.	3	Greger HG732 (WU)	Fiji	AY695570	-	-
A. yzermannii Boerl & Koord.		Pannell 1108 (FHO)	Peninsular Malaysia	KF212144	-	-
Lansium domesticum Correa		Chase 2113 (K)	Java	AY695586	-	-
Lansium cf. membranaceum (Kosterm.) Mabb.		Pannell 1934 (FHO)	Sumatra	DQ861611	-	-
Reinwardtiodendron celebicum Koord.		Nooteboom, H.P. 5973 (L)	Moluccas	KF212173	-	-
Reinwardtiodendron cinereum (Hiern) Mabb.	1	KEP F.R.I26877 (K)	Malaysia	AY695588	-	-
Reinwardtiodendron cinereum (Hiern) Mabb.	2	Mabberley and Pannell 1987 (FHO)	Peninsular Malaysia	KF212174	-	-
Reinwardtiodendron cinereum (Hiern) Mabb.	3	Mabberley and Pannell 1988 (FHO)	Peninsular Malaysia	KF212175	-	-
Reinwardtiodendron humile (Hassk.) Mabb.		Trichon VT641 (FHO)	Sumatra	DQ861612	-	-
Reinwardtiodendron kinabaluense (Kost.) Mabb.		Lamb ALFB112/87 (K)	Sabah	AY695589	-	-
Outgroup - Aglaieae						
Aphanamixis borneensis (Nig.) Herr.	1	Mabberley 1693 (FHO)	Sabah	KF212170	-	-
Aphanamixis borneensis (Nig.) Herr.	2	Beaman 8208 (K)	Sabah	AY695583	-	-
Aphanamixis polystachya (Wall.) R.N.Parker		Middleton et al. 1329 (K)	Thailand	KF212171	-	-
Aphanamixis sumatrana (Miq.) Ridl.		Trichon VT1085 (FHO)	Sumatra	KF212172	-	-
Sphaerosacme decandra (Wall.) Pennington		Williams and Stainton 8533 (K)	Nepal	AY695590	-	-
